# Predictive value of systemic immune-inflammation index in patients with diabetes mellitus: a systematic review and meta-analysis

**DOI:** 10.3389/fendo.2025.1617814

**Published:** 2025-09-23

**Authors:** Yujie Dong, Maoyuan Tang, Yanmei Xu, Qiyan Peng

**Affiliations:** ^1^ School of Nursing and Health Care, Leshan Vocational and Technical College, Leshan, Sichuan, China; ^2^ Department of Urology, Hospital of Traditional Chinese Medicine Leshan, Leshan, Sichuan, China

**Keywords:** SII, DM, prognosis, meta-analysis, mortality

## Abstract

**Background:**

Emerging evidence suggests an association between the systemic immune-inflammation index (SII) and the prognostic outcomes in individuals diagnosed with diabetes mellitus (DM). However, existing research presents inconsistent findings.

**Methods:**

PubMed, Embase, Web of Science, and the Cochrane Library from their inception to December 9, 2024, were retrieved to identify studies that scrutinized the interaction between SII and prognostic outcomes in DM patients. Primary outcomes included diabetic nephropathy (DN), diabetic retinopathy (DR), all-cause mortality, cardiovascular mortality, peripheral artery disease(PAD), and kidney mortality. Data were analyzed using odds ratios (ORs) or standardized mean differences (SMDs) with 95% confidence intervals (CIs). To ensure the robustness of results and uncover any underlying sources of heterogeneity, sensitivity analyses and subgroup analyses were also carried out.

**Results:**

Following a comprehensive literature search and screening, the meta-analysis incorporated 22 studies involving 85,796 patients. Categorical variable analysis revealed that elevated SII levels were correlated with a markedly increased risk of DN [OR = 1.94, 95% CI (1.02, 3.67), P = 0.04], all-cause mortality [OR = 1.38, 95% CI (1.14, 1.67), P = 0.001], and cardiovascular mortality [OR = 1.55, 95% CI (1.12, 2.16), P = 0.009] compared to those with lower SII levels. Furthermore, continuous variable analysis also indicated a significant link between SII levels and the incidence of DN [SMD = 4.56, 95% CI (1.44, 7.67), P = 0.004] and DR [SMD = 2.70, 95% CI (1.77, 3.64), P < 0.0001].

**Conclusion:**

SII serves as a reliable and profoundly meaningful biomarker in forecasting prognostic outcomes and assessing mortality risks among DM patients. However, given the limited quantity and quality of the studies included, these findings remain to be further validated.

**Systematic Review Registration:**

https://www.crd.york.ac.uk/prospero/, identifier CRD42024575794.

## 1 Introduction

Diabetes mellitus (DM) is a highly prevalent chronic metabolic disorder, with its global prevalence rising at an alarming rate. This escalating trend poses a significant burden on public health. The International Diabetes Federation (IDF) reports that an estimated 537 million adults across the globe suffered from DM as of 2021, a figure projected to soar to 783 million by 2045 (1). DM not only leads to a heavy burden of disease but is also associated with multiple serious complications, such as cardio-vascular disease (CVD), diabetic nephropathy (DN), diabetic retinopathy (DR), and diabetic peripheral artery disease (PAD) (2). These complications severely diminish patients’ well-being and significantly increase healthcare costs and mortality rates. For example, DN is the primary etiology of end-stage kidney disease (ESKD), with over 40% of ESKD patients worldwide having DM (3). Moreover, DR is the most prevalent cause of vision loss among the working-age population, while PAD may lead to severe limb dysfunction and even amputation. Despite substantial advances in glycemic control, complication prevention, and treatment, DM and its associated complications remain major global health challenges in terms of high morbidity and mortality. Conventional treatment methods, including lifestyle modifications, pharmacological interventions, and insulin therapy, are effective in controlling blood glucose levels but have limited efficacy in preventing and managing complications.

In recent years, with advances in understanding the pathophysiological mechanisms of DM, inflammation is increasingly investigated as a pivotal factor in both the onset and progression of DM as well as its associated complications. Study shows that chronic inflammation plays a role in the pathogenesis of DM and is also strongly linked to the onset and progression of DM-related complications (3). Therefore, identifying reliable biomarkers that can predict the risk of diabetic complications at an early stage has become a critical focus in improving the prognostic outcomes of DM patients. Systemic Immune-Inflammation Index (SII) is a composite inflammatory marker calculated using the formula: SII = platelet count × neutrophil count/lymphocyte count (4). SII integrates changes in multiple inflammatory cell types, so that it offers a comprehensive snapshot of the body’s inflammatory status. In recent years, SII has shown significant prognostic potential in various diseases, including cancer, cardio-vascular diseases, and infectious diseases (5–7). Its application in the field of DM has also gained increasing attention. Studies have demonstrated that SII levels are closely bound up with the inflammatory status of DM patients and may be related to the onset and progression of diabetic complications (8). For instance, a particular study has reported that individuals with DN exhibit considerably elevated SII levels compared to those without DN. This suggests that SII may be used as an early predictive marker for DN (9). Additionally, SII has been used to assess the severity and progression risk of DR (10).

Although prior research has explored the potential utility of SII in DM and its complications, current evidence remains inconsistent. Differences in SII calculation methods and reference ranges across studies may affect its applicability in different populations. Furthermore, the relationship between SII and diabetic complications requires further validation, particularly in patients of different ethnicities, genders, and disease stages (11, 12). Moreover, most existing studies are either cross-sectional or small-scale prospective studies, with a notable absence of large-scale, multicenter randomized controlled trials (RCTs) or long-term follow-up studies. Therefore, a systematic review and meta-analysis of the prognostic impact of SII on DM patients is essential to integrate existing evidence and clarify the clinical utility of SII. Such analysis will also provide a solid basis for future clinical research and practice. As a result, this study is designed to sum up the prognostic significance of SII in DM patients, explore its potential applications in different diabetic complications, and offer insights for later research and clinical practice.

## 2 Materials and methods

### 2.1 Literature search strategy

This study adhered to the guidelines set forth in the Preferred Reporting Items for Systematic Reviews and Meta-Analyses (PRISMA 2020) statement. The study protocol has been registered in the International Prospective Register of Systematic Reviews (PROSPERO: CRD42024575794). DYJ and TMY independently developed the search strategy by combining MeSH terms and keywords. Databases like PubMed, Embase, Web of Science, and the Cochrane Library were retrieved, spanning from the inception of each database to December 9, 2024. Broad terms such as “Diabetes Mellitus,” “Diabetes,” “DM,” “systemic immune inflammation index,” and “SII” were applied. The literature search strategy is detailed in **Supplementary Table S1**.

### 2.2 Study selection

Any study that fulfilled the following criteria was selected for inclusion in the analysis:

1. Patients had confirmed diagnosis of DM.
2. The study investigated the influence of SII on the prognosis of DM patients.
3. The study provided odds ratios (ORs) with 95% confidence intervals (CIs) or standardized mean differences (SMDs), either directly extracted or derived from available data.
4. Patients were divided into high SII versus low SII group, or poor versus favorable prognosis groups based on predefined thresholds.

Exclusion criteria were detailed as follows:

1. Studies focusing on animal experiments, reviews, comments, conference abstracts, case reports, or letters.
2. Studies lacking sufficient data to calculate OR + 95%CI or SMDs.
3. Studies with no data on SII and/or DM prognosis.
4. Studies with duplicate or overlapping data.

DYJ and TMY separately screened the titles and abstracts of studies retrieved from the databases. Eligible full-text articles were then downloaded and evaluated. Discrepancies during the selection were settled through mutual agreement.

### 2.3 Data extraction

DYJ and TMY also carried out data extraction independently. Any discrepancies were resolved through mutual agreement among all co-authors. Extracted information encompassed the first author’s name, publication year, country (study location), study type, sample size, patient age, gender, BMI, FBG, SII cutoff values, and relevant outcome indicators (e.g., DN, DR, all-cause mortality, cardiovascular mortality, CVD, PAD, diabetic macular edema [DME] with SMD, metabolic syndrome, and kidney mortality).

### 2.4 Quality assessment

The risk of bias in the studies selected for the meta-analysis was evaluated using the Newcastle-Ottawa Scale (NOS). Any study with a score of ≥6 was categorized as high-quality (13).

### 2.5 Statistical analysis

Categorical variables were integrated using ORs with 95% CIs, while continuous variables were pooled using SMDs with 95% CIs to dig into the link between SII and the prognostic outcomes of DM patients. Heterogeneity was quantified employing the well-established Cochran’s Q test and Higgins I² statistic. The presence of significant heterogeneity was indicated by I² > 50% or P < 0.1. A random-effects model was used for all analyses. To disentangle the origin of heterogeneity and assess the robustness of results, subgroup analyses were conducted based on SII cutoff values, region, sample size, and age. Sensitivity analysis was performed using a leave-one-out method. Potential publication bias was assessed via funnel plots and Egger’s test, with statistical significance defined as P < 0.05. For outcomes with potential publication bias, the trim-and-fill method was used to assess the impact of such bias on the results. All statistical analyses were carried out using Review Manager 5.4 and STATA 15.0 software.

## 3 Results

### 3.1 Literature screening process and results

**Figure 1:**
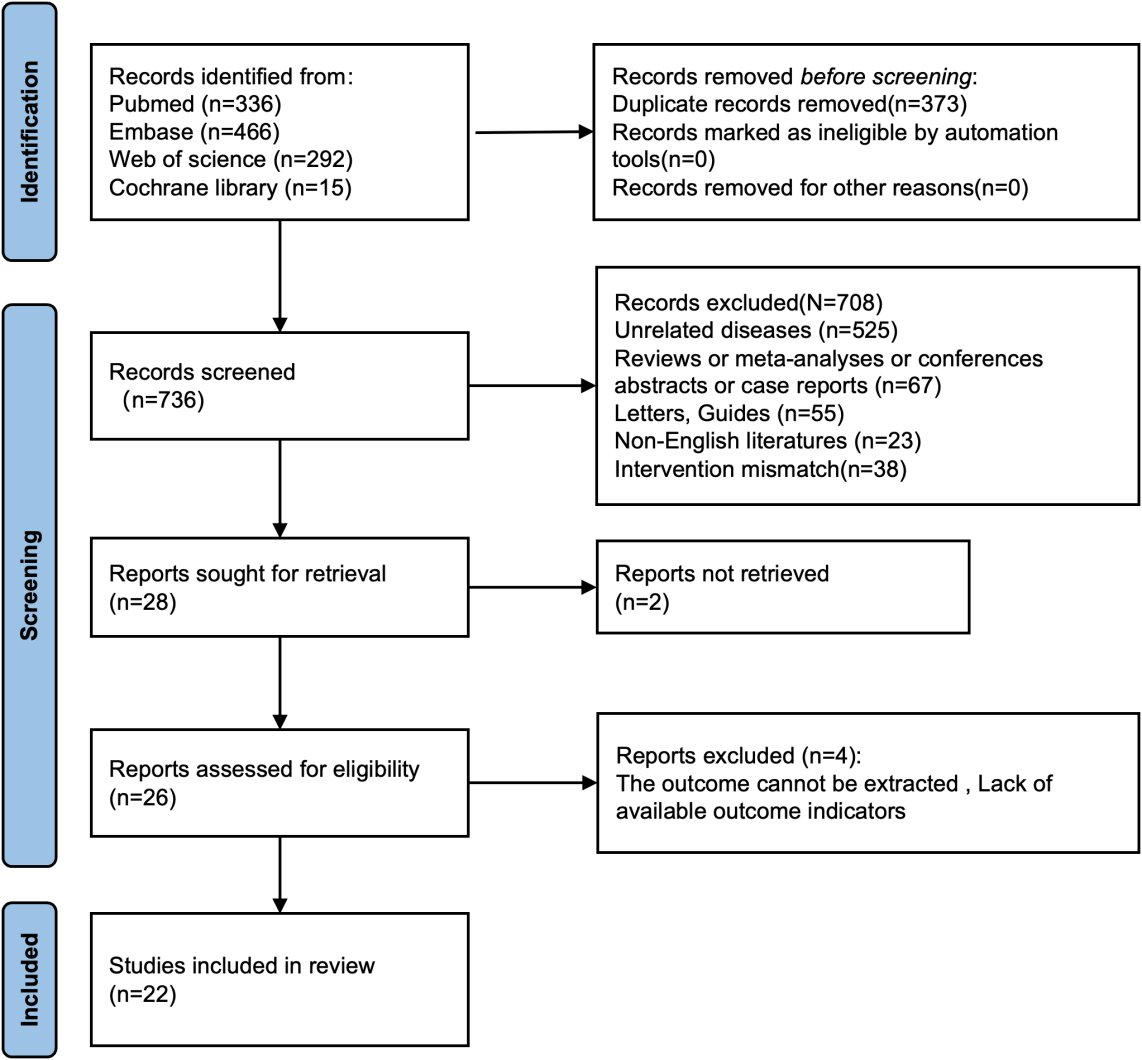
Flowchart for selection of studies included in this meta-analysis based on PRISMA guidelines.

Initially, there were altogether 1,109 relevant studies identified from PubMed, Embase, Web of Science, and the Cochrane Library. After the removal of 373 duplicate studies, 708 studies were excluded during the screening of titles and abstracts. The full texts of 28 studies were then reviewed. Two studies were excluded due to inaccessible full texts. Four studies were excluded because of insufficient data on DM-related outcomes. Consequently, 22 articles involving 85,796 patients were incorporated in the final meta-analysis (**Figure 1**).

### 3.2 Study characteristics and quality assessment

The 22 included articles were published between 2022 and 2024. Of these, 11 studies (8, 14–23) were conducted in Asia, 7 studies (9, 24–29) in the Americas, and 4 studies (10, 30–32) in Europe. Notably, 7 studies (8, 10, 16, 24, 26, 27, 29) included 2 comparison groups each, and 3 studies (19, 22, 28) included 3 comparison groups each, resulting in a total of 35 comparison groups. Among these, 16 were retrospective cohort studies (8–10, 14–23, 26, 30, 32), and 6 were prospective studies (24, 25, 27–29, 31). The mean patient age exceeded 60 years in 7 studies (9, 10, 19–21, 25, 30), while 13 studies (8, 14, 16, 18, 22–24, 26–29, 31, 32) involved patients aged ≤60 years. 2 studies (15, 17) did not provide age data. In all studies, SII served as a prognostic risk factor for DM, with cutoff values varying across studies. SII was defined as ≥500 in 6 studies (10, 26–30), and <500 in 5 studies (8, 9, 18, 24, 31). The remaining studies failed to provide specific SII cutoffs.

The prognosis of DM varied across the 22 studies: 6 studies (24–29) reported the relationship between SII and all-cause mortality; 5 studies (24, 26–29) assessed the connection between SII and cardiovascular mortality; 1 study (28) examined the association between SII and kidney mortality; 1 study (20) investigated the relation of SII to mortality following below-knee amputation; 6 studies (9, 16, 17, 19, 22, 23) explored the relationship between SII and DN(8 comparison groups); 5 studies (8, 10, 18, 21, 22) analyzed the association between SII and DR (7 comparison groups); 3 studies (16, 30, 32) assessed the correlation between SII and CVD; 2 studies (15, 22) investigated the link between SII and PAD; 1 study (31) probed into the relationship between SII and metabolic syndrome; 1 study (14) examined the association between SII and serous macular detachment secondary to diabetic macular edema.

The NOS-based quality assessment showed that all included studies achieved a score of at least 6, indicating high quality and low risk of bias.

The characteristics and quality assessment of the included studies are set out in **Table 1**. Detailed characteristics of included studies are presented in **Supplementary Table S2**.

**Table 1:** Summary of general characteristics of included studies.

Author+year	Region	Study design	Population^1^	No. of patients	SII cut-off^2^	Outcome	Quality score
Alhalwani (21) 2023	Saudi Arabia	Retrospective	T2DM	185	NA	DR	7
Chen (24) 2023a	United States	Prospective	T2DM	8668	NA	Cardiovascular mortality	7
Chen (24) 2023b	United States	Prospective	T2DM	8668	NA	All-cause mortality	7
Dascalu (10) 2023a	Romania	Retrospective	T2DM	129	763.8	Non-proliferative DR	7
Dascalu (10) 2023b	Romania	Retrospective	T2DM	129	763.8	Proliferative DR	7
Duman (17) 2023	Turkey	Retrospective	T2MD	539	NA	DN	7
Fajkic (31) 2024	Bosnia and Herzegovina	Prospective	T2DM	80	408.57	Metabolic syndrome	7
Gao (8) 2024a	China	Retrospective	T2DM	141	260.65	Non-proliferative DR	7
Gao (8) 2024b	China	Retrospective	T2DM	141	260.65	Proliferative DR	7
Guo (9) 2022	United States	Retrospective	T2MD	3937	445.21	DN	7
Li (22) 2024a	China	Retrospective	T2DM	1058	NA	DN	6
Li (22) 2024b	China	Retrospective	T2DM	1058	NA	DR	6
Li (22) 2024c	China	Retrospective	T2DM	1058	NA	PAD	6
Li (25) 2024	United States	Prospective	DM	983	NA	All-cause mortality	7
Liu (23) 2024	China	Retrospective	T2DM	234	659.09	DN	7
Mariaca (32) 2024	Spain	Retrospective	T1DM	602	NA	CVD	7
Meng (26) 2024a	United States	Retrospective	DM	4972	983.5714	Cardiovascular mortality	7
Meng (26) 2024b	United States	Retrospective	DM	4972	983.5714	All-cause mortality	7
Muresan (30) 2023	Romania	Retrospective	T2DM	198	615.91	CVD	7
Özata Gündoğdu (14)2022	Turkey	Retrospective	DM	120	NA	serous macular detachment secondary to diabetic macular edema	9
Song (15) 2023	China	Retrospective	T2DM	434	NA	PAD	7
Suvarna (16) 2023a	India	Retrospective	T2DM	300	NA	DN	6
Suvarna (16) 2023b	India	Retrospective	T2DM	300	NA	CVD	6
Tang (27) 2024a	United States	Prospective	DM	45454	963.1	Cardiovascular mortality	7
Tang (27) 2024b	United States	Prospective	DM	45454	963.1	All-cause mortality	7
Wang (18) 2023	China	Retrospective	T2DM	500	419.5762	DR	7
Yan (19) 2023a	China	Retrospective	T2DM	1922	NA	DN stages 1–2 Alb	7
Yan (19) 2023b	China	Retrospective	T2DM	1922	NA	DN stage 3 Alb+ DN -non-Alb	7
Yan (19) 2023c	China	Retrospective	T2DM	1922	NA	DN	7
Yang (28) 2023a	United States	Prospective	T2DM	8697	702.6	Kidney mortality	7
Yang (28) 2023b	United States	Prospective	T2DM	8697	702.6	Cardiovascular mortality	7
Yang (28) 2023c	United States	Prospective	T2DM	8697	702.6	All-cause mortality	7
Yilmaz (20) 2023	Turkey	Retrospective	DM	231	NA	Mortality after below-knee amputation	7
Zhang (29) 2024a	United States	Prospective	DM	6412	692.13	Cardiovascular mortality	7
Zhang (29) 2024b	United States	Prospective	DM	6412	692.13	All-cause mortality	7

^1^The study using “DM” included a mixed population and did not clearly distinguish between T1DM and T2DM.

^2^The original study determined the optimal threshold of SII for predicting prognosis in DM patients based on the receiver operating characteristic (ROC) curve.

DR, Diabetic retinopathy; DN, Diabetic nephropathy; CVD, Cardiovascular disease; PAD, Peripheral artery disease; DM, Diabetes Mellitus; T1DM, type 1 Diabetes Mellitus; T2DM, type 2 Diabetes Mellitus; SII, systemic immune-inflammatory index; NA, Data not reported in the source manuscript.

### 3.3 Meta-analysis results

#### 3.3.1 Analysis of SII and DM-related outcomes

##### 3.3.1.1 Correlation between SII and diabetic nephropathy

**Figure 2:**
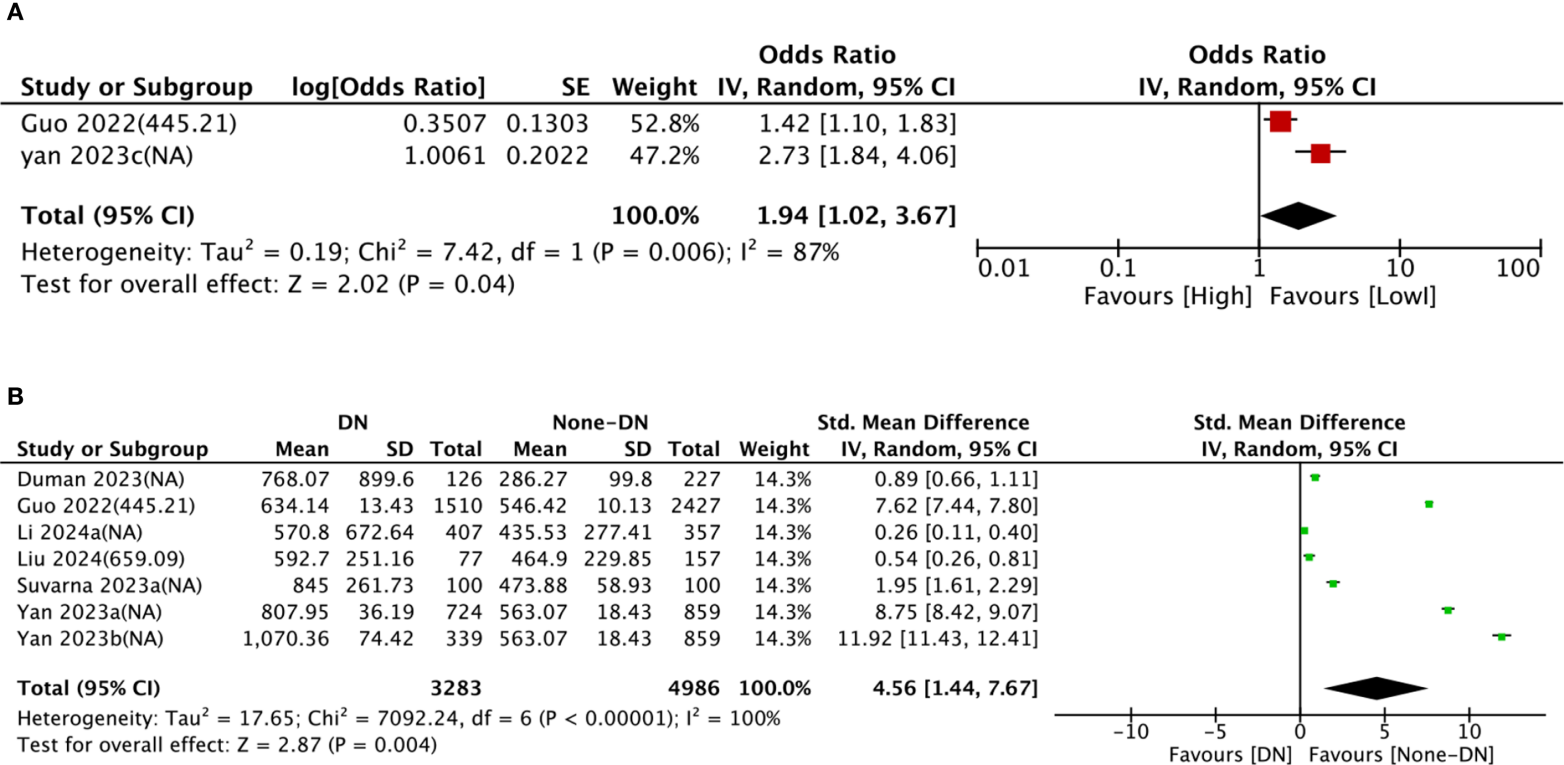
Forest plots depicting the association between elevated SII and risk of DN. **(A)** Categorical variable analysis: Odds ratios (OR) and 95% confidence intervals (CI) for high vs. low SII groups. Random-effects model was used due to significant heterogeneity (I² = 87%). **(B)** Continuous variable analysis: Standardized mean differences (SMD) and 95% CI comparing SII levels in DN group vs. non-DN group (SMD > 0.8 indicates large effect size). Random-effects model was used due to significant heterogeneity (I² = 100%). SII cutoff (unit: ×10^9^/L) values used in each study are indicated.

Nine comparison groups explored the relationship between SII and DN. The pooled analysis of ORs with 95% CIs from two comparison groups revealed significant heterogeneity (I^2^ = 87%, P = 0.006). Consequently, a random-effects model was leveraged. The results indicated that patients with higher SII had a higher incidence of DN (OR = 1.94, 95% CI: 1.02 - 3.67, P = 0.04) (**Figure 2A**). The pooled analysis of SMDs from seven comparison groups also showed significant heterogeneity (I^2^ = 100%, P < 0.0001). A random-effects model was adopted. Patients in the DN group exhibited significantly higher SII levels compared to the non-DN group (SMD = 4.56, 95% CI: 1.44–7.67, P = 0.004) (**Figure 2B**).

To dig into the causes of heterogeneity, subgroup analysis was carried out based on age, SII cut-off, sample size, region, and study design, as detailed in **Table 2**. In comparison groups with participants aged ≥60 years (SMD = 9.42, 95% CI: 7.35–11.49, P < 0.0001), DN group showed significantly higher SII levels than non-DN group, whereas no significant relationship between SII and DN was observed in comparison groups with participants aged <60 years (SMD = 0.90, 95% CI: −0.00 to 1.81, P = 0.05). Subgroup analyses by sample size and region both demonstrated significantly higher SII levels in DN groups than in non-DN groups. The forest plot of the subgroup analysis is illustrated in **Supplementary Figure S1**.

**Table 2:** Subgroup analysis of SII and diabetes outcomes^1^.

Subgroup	Diabetic nephropathy				Diabetic retinopathy				All-cause mortality				Cardio-vascular mortality			
	Study	SMD [95%CI]	*P* value	*I* ^2^	Study	SMD [95%CI]	*P* value	*I* ^2^	Study	OR [95%CI]	*P* value	*I* ^2^	Study	OR [95%CI]	*P* value	*I* ^2^
*Total*	7	4.56 [1.44-7.67]	0.004	100%	7	2.70 [1.77-3.64]	<0.0001	98%	6	1.38 [1.14-1.67]	0.001	87%	5	1.55[1.12-2.16]	0.009	85%
Mean/median age																
≥60y	3	9.42[7.35-11.49]	<0.0001	99%	3	0.15[-0.13-0.43]	0.3	39%	5	1.38[1.11-1.73]	0.004	88%				
<60y	3	0.90[-0.00-1.81]	0.05	98%	4	5.57[3.93-7.22]	<0.0001	99%	1	1.36 [1.10-1.68]	0.005	NA				
SII cut-off																
≥500					2	0.26[-0.19-0.71]	0.25	52%	4	1.41[1.06-1.87]	0.02	89%	4	1.55[1.04-2.30]	0.03	86%
<500					3	8.65[-0.93-18.23]	0.08	99%	1	1.33[1.10-1.61]	0.003	NA	1	1.59[1.11-2.28]	0.01	NA
Sample size																
≥500	4	7.13[1.98-12.29]	0.007	100%	2	0.45[-0.01-0.91]	0.06	93%								
<500	3	1.12[0.38-1.85]	0.003	95%	5	4.76[2.56-6.97]	<0.0001	99%								
Region																
Asia	5	4.68[0.79-8.56]	0.02	100%	5	4.05[2.80-5.31]	<0.0001	99%								
America	1	7.62[7.44-7.80]	<0.0001	NA												
Europe	1	0.89[0.66-1.11]	<0.0001	NA	2	0.26[-0.19-0.71]	0.25	52%								
Study design																
Prospective									5	1.33[1.09-1.61]	0.004	83%	4	1.44[1.04-1.99]	0.03	80%
Retrospective									1	1.36 [1.10-1.68]	0.005	NA	1	2.05[1.42-2.96]	0.0001	NA

^1^Caution: Interpretations of subgroup analysis findings, particularly those involving few comparison groups (e.g., n <3), require careful consideration, as estimates may be unstable and less reliable.

##### 3.3.1.2 Correlation between SII and DR

**Figure 3:**
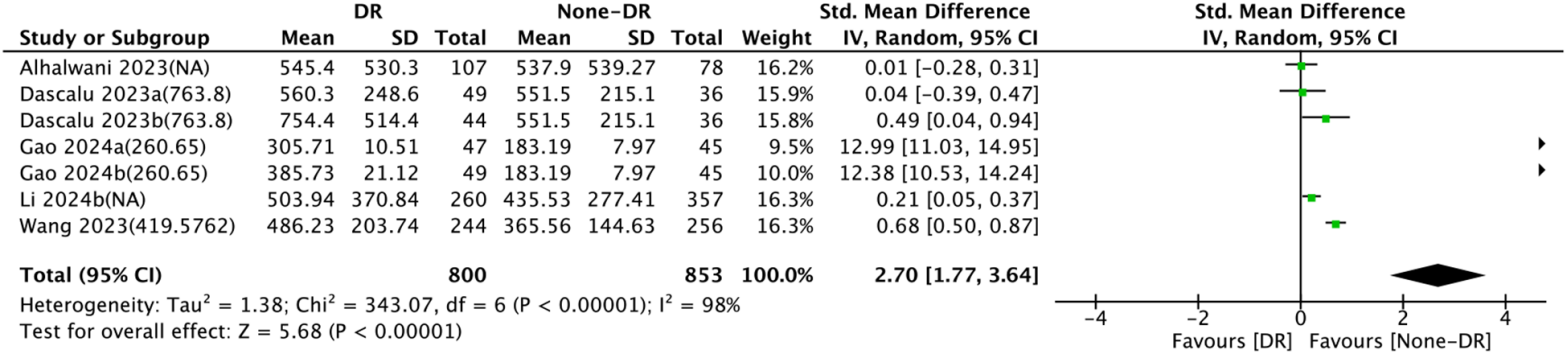
Forest plots depicting the association between elevated SII and risk of DR. Continuous variable analysis: Standardized mean differences (SMD) and 95% CI comparing SII levels in DR group vs. non-DR group (SMD > 0.8 indicates large effect size). Random-effects model was used due to significant heterogeneity (I² = 98%). SII cutoff (unit: ×10^9^/L) values used in each study are indicated.

Seven comparison groups examined the association between SII and DR. The pooled analysis of SMDs demonstrated substantial heterogeneity (I² = 98%, P < 0.0001). A random-effects model was leveraged. The results unraveled that patients in the DR group had considerably higher SII levels compared to the non-DR group (SMD = 2.70, 95% CI: 1.77–3.64, P < 0.0001) (**Figure 3**).

First, subgroup analysis by age showed that in comparison groups with participants aged <60 years (SMD = 5.57, 95% CI: 3.93–7.22, P < 0.0001), SII levels were substantially increased in the DR group relative to the non-DR group. Nonetheless, no significant relationship between SII and DR was observed in comparison groups with participants aged ≥60 years (SMD = 0.15, 95% CI: −0.13 to 0.43, P = 0.3). Second,

subgroup analysis based on sample size revealed that in comparison groups with <500 participants (SMD = 4.76, 95% CI: 2.56–6.97, P < 0.0001), SII levels were markedly elevated in the DR group compared to the non-DR group, whereas no significant relationship between SII and DR was found in comparison groups with ≥500 participants (SMD = 0.45, 95% CI: −0.01 to 0.91, P = 0.06). Subgroup analysis by region indicated that in Asian comparison groups (SMD = 4.05, 95% CI: 2.80–5.31, P < 0.0001), SII levels were significantly higher in the DR group than that in non-DR group, whereas no significant association between SII and DR was observed in European comparison groups (SMD = 0.26, 95% CI: −0.19 to 0.71, P = 0.25). Similarly, subgroup analysis based on SII cut-off values revealed elevated SII levels in DR groups versus non-DR groups. The details are set out in **Table 2**. The forest plot of the subgroup analysis is illustrated in **Supplementary Figure S2**.

##### 3.3.1.3 Correlation between SII and all-cause mortality

**Figure 4:**
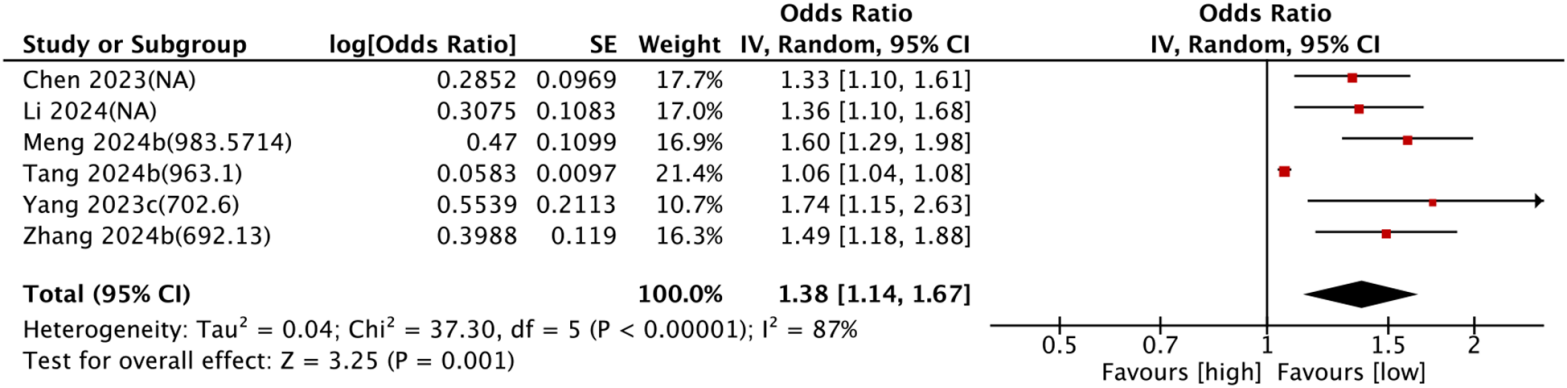
Forest plots depicting the association between elevated SII and All-cause mortality. Categorical variable analysis: Odds ratios (OR) and 95% confidence intervals (CI) for high vs. low SII groups. Random-effects model was used due to significant heterogeneity (I² = 87%). SII cutoff (unit: ×10^9^/L) values used in each study are indicated.

Six comparison groups examined the link between SII and all-cause mortality. Pooled analysis of ORs with 95% CIs revealed significant heterogeneity (I^2^ = 87%, P < 0.0001). A random-effects model was leveraged, and higher SII levels were associated with a significantly increased risk of all-cause mortality (OR = 1.38, 95% CI: 1.14–1.67, P = 0.001) (**Figure 4**).

Subgroup analysis consistently revealed the prognostic potential of SII for all-cause mortality. The details are set out in **Table 2**.

##### 3.3.1.4 Correlation between SII and cardiovascular mortality

**Figure 5:**
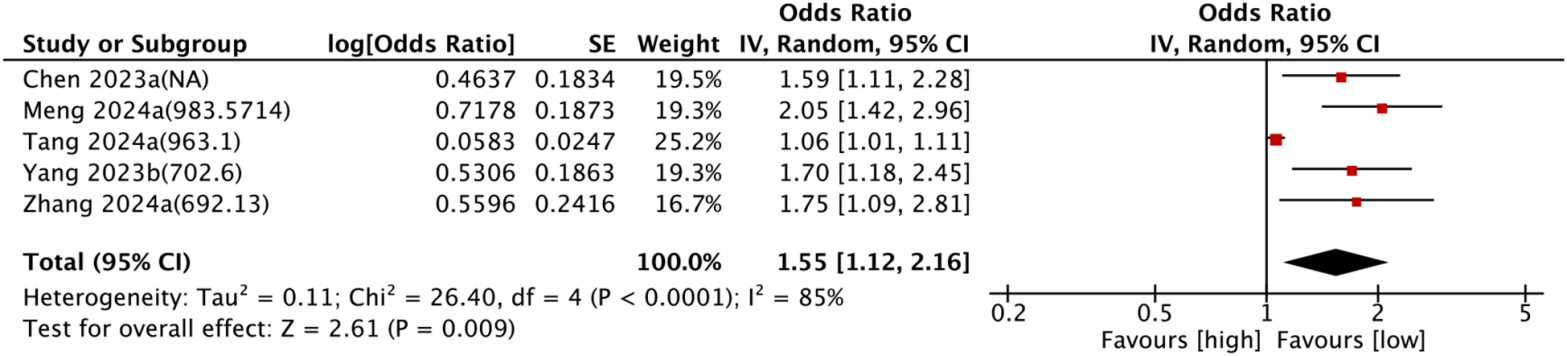
Forest plots depicting the association between elevated SII and Cardiovascular mortality. Categorical variable analysis: Odds ratios (OR) and 95% confidence intervals (CI) for high vs. low SII groups. Random-effects model was used due to significant heterogeneity (I² = 85%). SII cutoff (unit: ×10^9^/L) values used in each study are indicated.

Five comparison groups assessed the relationship between SII and cardiovascular mortality. Pooled analysis of ORs with 95% CIs revealed significant heterogeneity (I^2^ = 85%, P < 0.0001). A random-effects model was employed, and the results unveiled that higher SII levels were associated with a significantly increased risk of cardiovascular mortality (OR = 1.55, 95% CI: 1.12–2.16, P = 0.009) (**Figure 5**).

Subgroup analysis consistently confirmed the prognostic value of SII for cardiovascular mortality. The details are set out in **Table 2**.

##### 3.3.1.5 Correlation between SII and CVD

**Figure 6:**
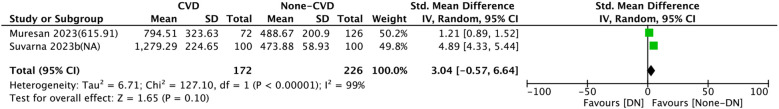
Forest plots depicting the association between elevated SII and CVD. Continuous variable analysis: Standardized mean differences (SMD) and 95% CI comparing SII levels in CVD group vs. non-CVD group (SMD > 0.8 indicates large effect size). Random-effects model was used due to significant heterogeneity (I² = 99%). SII cutoff (unit: ×10^9^/L) values used in each study are indicated.

Two comparison groups explored the relationship between SII and CVD. Pooled analysis of SMDs revealed significant heterogeneity (I² = 99%, P < 0.0001). A random-effects model was leveraged for data analysis. No significant connection was observed between SII and cardiovascular disease (SMD = 3.04, 95% CI: −0.57 to 6.64, P > 0.05) (**Figure 6**).

##### 3.3.1.6 Correlation between SII and PAD

**Figure 7:**
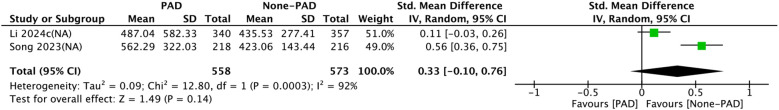
Forest plots for the association between SII and PAD. Continuous variable analysis: Standardized mean differences (SMD) and 95% CI comparing SII levels in PAD group vs. non-PAD group (SMD < 0.5 indicates small effect size). Random-effects model was used due to significant heterogeneity (I² = 99%). SII cutoff (unit: ×10^9^/L) values used in each study are indicated.

Two comparison groups investigated the relationship between SII and PAD. Pooled analysis of SMDs showed significant heterogeneity (I² = 99%, P=0.0003). According to the meta-analysis by a random-effects model, no significant correlation was observed between SII and PAD (SMD = 0.33, 95% CI: −0.10 to 0.76, P > 0.05) (**Figure 7**).

##### 3.3.1.7 SII and other diabetic outcomes

Özata et al. (14) included 120 patients from 2015 to 2020 in their study published in 2015, and concluded that significantly elevated SII levels existed in DME patients, particularly in advanced cases (P = 0.001).

Fajkic et al. (31) included 80 patients over two years in their study published in 2024, and reported significantly higher SII levels in T2DM patients with Metabolic Equivalents (Mets) (P < 0.001).

Yang et al. (28) included 8697 patients from 1999 to 2018 in their study published in 2023, and concluded that no close correlation existed between SII and kidney mortality (HR = 0.9, 95% CI: 0.11–7.14, P = 0.545).

### 3.4 Sensitivity analysis

**Figure 8:**
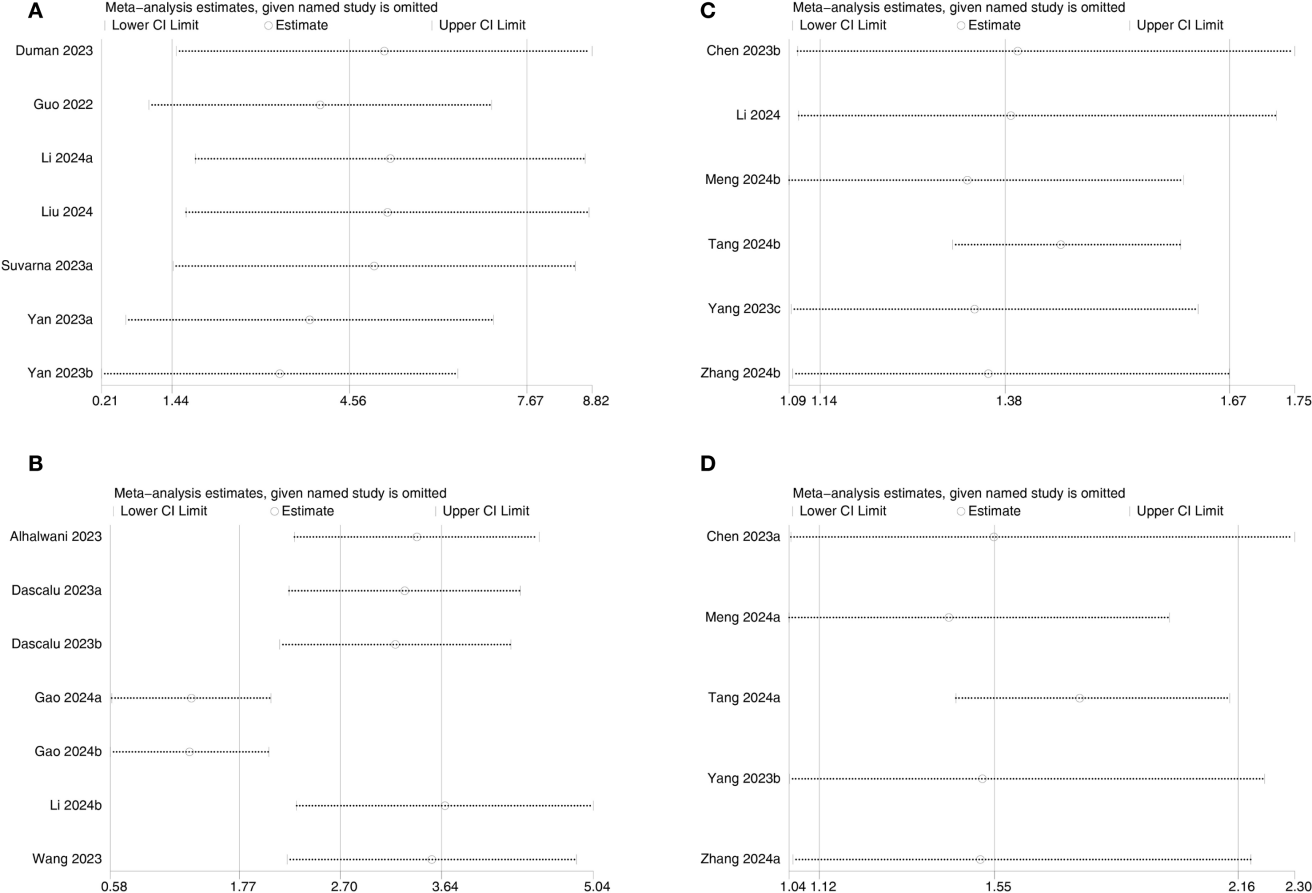
Sensitivity analysis of **(A)** DN, **(B)** DR, **(C)** All-cause mortality, and **(D)** Cardiovascular mortality.

Sensitivity analysis was executed for outcomes reported in ≥2 comparison groups by sequentially excluding individual comparison groups. Results showed no significant changes in results for DN (**Figure 8A**), DR (**Figure 8B**), all-cause mortality (**Figure 8C**), and cardiovascular mortality (**Figure 8D**), confirming the reliability of the findings.

### 3.5 Publication bias

**Figure 9:**
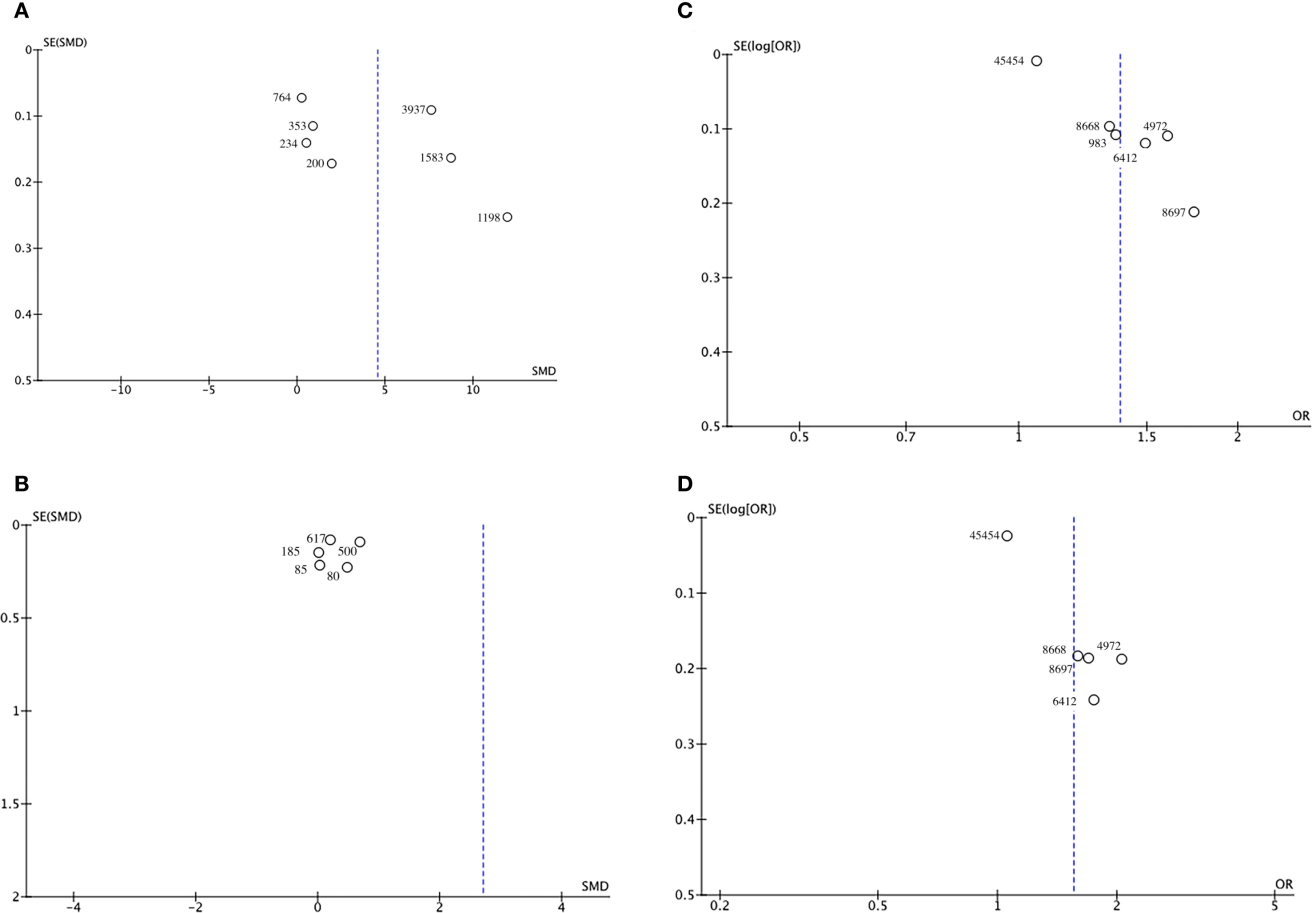
Funnel plot for the evaluation of publication bias for **(A)** DN, **(B)** DR, **(C)** All-cause mortality, and **(D)** Cardiovascular mortality.

Funnel plots and Egger’s regression test were employed to assess publication bias. The funnel plots were symmetrical for DN (continuous variable) (**Figure 9A**) and all-cause mortality (categorical variable) (**Figure 9C**), suggesting a low likelihood of publication bias. In contrast, the funnel plots were asymmetrical for DR (continuous variable) (**Figure 9B**) and cardiovascular mortality (categorical variable) (**Figure 9D**), indicating possible publication bias.

**Figure 10:**
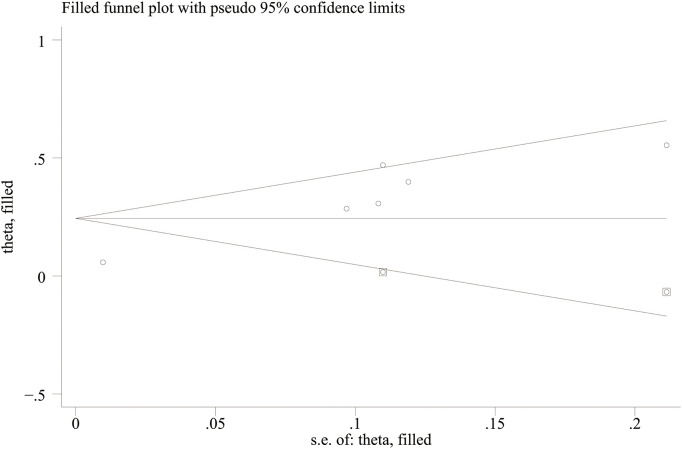
Trim-and-fill adjustment for the association between SII and all-cause mortality.

**Figure 11:**
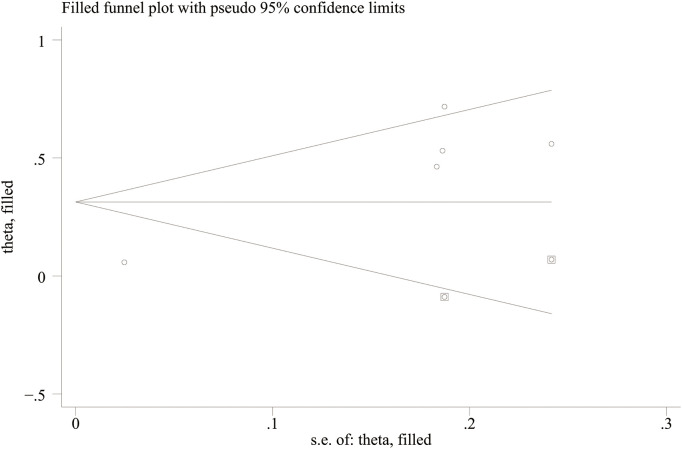
Trim-and-fill adjustment for the association between SII and cardiovascular mortality.

Egger’s regression results were consistent with these findings. No substantial evidence of publication bias was identified for DN (continuous variable) (P = 0.344). However, significant small-study effects were identified for all-cause mortality (categorical variable) (P = 0.001), cardiovascular mortality (categorical variable) (P = 0.005), and DR (continuous variable) (P = 0.05), suggesting potential publication bias. Publication bias was evaluated for its impact on all-cause mortality (categorical variable) and cardiovascular mortality (categorical variable) using the trim-and-fill method. The results showed that the statistical significance of all-cause mortality (categorical variable) (OR = 1.28, 95% CI: 1.10–1.49; **Figure 10**) and cardiovascular mortality (categorical variable) (OR = 1.37, 95% CI: 1.07–1.74; **Figure 11**) remained unchanged after adjustment. This implies that publication bias did not materially affect the findings. Publication bias was not be assessed for other outcomes due to insufficient comparison groups (N < 3).

## 4 Discussion

SII is a novel immune-inflammation index that integrates platelet count, neutrophil count, and lymphocyte count to comprehensively reflect the immune function and inflammatory status of the body. Initially, the SII was employed as a prognostic indicator for patients suffering from various malignancies (including non-small cell lung cancer, gastric cancer, and rectal cancer) (8, 12, 14). With further research, it is used in the field of DM, particularly for elucidating the immune mechanisms underlying the development and progression of DM. It has been shown that DM patients often exhibit elevated levels of inflammatory markers, which are closely linked to chronic low-grade inflammation induced by insulin resistance (33). This chronic low-grade inflammation not only accelerates the progression of DM but also facilitates the occurrence of various complications, such as DN and DR, creating a vicious cycle. Recent studies suggest that anti-inflammatory therapies play an increasingly important role in the clinical management of DM. By mitigating inflammation, these therapies not only enhance glycemic control but also boost insulin secretion (34, 35). Therefore, as a simple and cost-effective immune-inflammation index, SII is promising to be used as a valuable tool for prognostic evaluation in DM patients.

This systematic review and meta-analysis evaluated the clinical utility of SII in DM patients by examining its relationship with DM-related complications and exploring potential underlying mechanisms. Data from 22 studies involving 85,796 participants were analyzed. Pooled results showed that higher SII levels were significantly associated with increased risks of DN, DR, all-cause mortality, and cardiovascular mortality. Sensitivity analyses confirmed the robustness of these associations. However, substantial heterogeneity among studies requires cautious interpretation and emphasizes the need for further validation.

A methodological challenge was observed in the discordance between symmetrical funnel plots and statistically significant Egger’s test results for all-cause mortality. This discrepancy may arise from several factors beyond publication bias, such as true clinical heterogeneity due to variations in population characteristics or SII measurement protocols, unmeasured confounding variables, or type I errors from multiple testings. These observations collectively emphasize the need to carefully consider both statistical and clinical heterogeneity when evaluating the prognostic value of SII in DM populations.

The analysis in this study indicated that DM patients with elevated SII levels, particularly those with DN, exhibited significantly higher SII levels than those without DN. This finding suggests that SII, as a comprehensive indicator of systemic immune-inflammatory status, may be vital in the development of diabetic complications. SII reflects systemic inflammatory activation by integrating changes in neutrophil, platelet, and lymphocyte ratios in peripheral blood. Chronic low-grade inflammation has been recognized as a key factor in promoting diabetic complications, particularly renal damage (9). DM patients often suffer from hyperglycemia and insulin resistance. The prolonged metabolic disturbances activate inflammatory responses, especially neutrophil and platelet activation. These responses contribute to endothelial dysfunction, ultimately damaging the tubular and glomerular structures (36). Such inflammatory responses promote the occurrence and progression of DN through multiple mechanisms, and SII, as a marker of immune-inflammatory status, effectively reflects the overall inflammatory burden.

Further subgroup analysis revealed a significant age-dependent association between SII and DN risk. In patients aged 60 years and above, elevated SII levels were strongly associated with higher DN incidence, whereas the association was markedly weaker in younger cohorts. This differential susceptibility appears to stem from fundamental age-related changes in renal pathophysiology. As the immune microenvironment evolves with aging, several interconnected factors amplify renal vulnerability: the gradual decline in tissue repair capacity parallels accumulating mitochondrial dysfunction and progressive impairment of regulatory T cell function. These senescent changes collectively enhance the kidney’s sensitivity to systemic inflammatory insults, as quantified by sustained SII elevation.

The pathophysiological cascade involves excessive neutrophil and platelet activation, the core components of SII. These cells promote endothelial injury, oxidative stress, and fibrotic remodeling, thereby driving DN progression. Particularly in elderly populations, age-related inflammatory susceptibility combined with impaired repair capacity makes SII a robust predictor of DN risk. It effectively captures this critical intersection of inflammation and renal senescence.

However, no similar association was observed in younger patients, possibly suggesting the presence of compensatory mechanisms. For instance, younger individuals may possess a stronger renal repair capacity and may mitigate inflammatory damage through autophagic pathways (37). These findings provide a basis for developing age-specific monitoring strategies. Dynamic SII monitoring may be beneficial for old DM patients, while SII should be combined with other biomarkers to enhance predictive sensitivity for younger populations (38).

The analysis of DR proved that patients diagnosed with DR exhibited much higher SII levels than those without DR. This suggests the potential clinical value of SII in the development of DR. DR, as one of the most prevalent microvascular complications of DM, is closely bound up with systemic inflammation. SII reflects the degree of inflammation by integrating neutrophil, platelet, and lymphocyte ratios. Thereby, it can sensitively reflect changes in the systemic immune status. Retinopathy development is thought to be significantly influenced by chronic inflammation, which is regarded as a crucial factor in this process (8). Specifically, elevated SII indicates an increase in inflammatory cells, particularly neutrophils and platelets, in peripheral blood. These cells affect retinal microvascular permeability and function through multiple pathways, thereby promoting the progression of DR. Previous studies have shown that neutrophil-platelet aggregation accelerates endothelial injury and microvascular damage, exacerbating the development of DR (39). Therefore, elevated SII may act as a biomarker for an increased risk of DR in DM patients.

Further subgroup analysis disclosed notable differences in SII’s predictive value in DR across regions and age groups. SII was significantly linked to DR in Asian subgroups (n=5) and younger patient subgroups (<60 years, n=4) but not in the smaller European subgroups (n=2) or older patient subgroups (≥60 years, n=3). These discrepancies may relate to genetic background and metabolic control. A recent genomic study reported a high prevalence of TLR4 gene polymorphisms in Asians. It may heighten inflammatory responses under hyperglycemic environments, contributing to retinal barrier disruption (40). Moreover, HbA1c control is generally poorer in Asian DM patients than in Western populations. The synergistic effect of hyperglycemia and inflammation may therefore amplify the predictive efficacy of SII (41). While inflammatory processes contribute to DR initiation and progression, hyperglycemia-induced retinal endothelial damage and pericyte loss remain the predominant drivers. These changes increase vascular permeability and promote neovascularization, further aggravated by the accumulation of advanced glycation end products (AGEs). In elderly patients, the direct detrimental effects of hyperglycemia may outweigh systemic inflammatory contributions, making SII less predictive. Moreover, DR in the elderly often features retinal ischemia and neurodegeneration, which are less responsive to systemic inflammatory activity compared with glomerular injury in DN. Consequently, the association between SII and DR may be weaker in the elderly population due to differences in the predominant disease mechanisms. By contrast, younger patients typically experience a more aggressive DR progression pattern, marked by rapid deterioration and a pronounced Th17/Treg imbalance. This immune dysregulation is partly captured by the neutrophil-to-lymphocyte ratio (NLR), a key component of SII (42). However, current studies using fixed SII cutoffs have not demonstrated significant predictive value. This suggests that the existing cutoffs may not be applicable to the risk assessment of DR. Future studies should develop dynamic threshold models that incorporate glycemic variability and baseline inflammatory levels to improve prediction accuracy. For instance, integrating SII with HbA1c variability may provide more precise predictive indicators (43).

The findings in this study further demonstrate a substantial increase in all-cause and cardiovascular mortality among patients with elevated SII levels. Additionally, they underscore the critical role of SII as an indicator of systemic immune-inflammatory status in predicting mortality in DM patients. Since CVD remains the primary cause of death in DM patients, SII may be promising in forecasting all-cause and cardiovascular mortality in DM and other chronic diseases. Earlier research has demonstrated a robust connection between elevated SII levels and the mortality risk in DM patients (26). Particularly, SII can serve as an early warning indicator of mortality to help clinicians develop more personalized treatment strategies. Neutrophil-derived myeloperoxidase (MPO) promotes the instability of atherosclerotic plaque through LDL oxidation, while platelet-activated CD40 ligand (CD40L) induces macrophages to secrete matrix metalloproteinases (MMPs), accelerating plaque rupture. Simultaneously, lymphopenia weakens immune surveillance, resulting in an increased risk of fatal arrhythmias (44, 45). These mechanisms collectively establish a plausible “inflammation-electrophysiology link”, mechanistically bridging the robust SII-mortality association observed in clinical outcomes. Increased all-cause mortality is generally attributable to the combined impact of various fatal factors, including infection, cardio-vascular events, and cancer. As an indicator of systemic immune-inflammatory responses, SII reveals prolonged immune activation and dysfunction, thus influencing the onset and progression of multiple diseases.

Furthermore, cardiovascular mortality is a major cause of death in DM patients. The development of CVD is closely connected to atherosclerosis, thrombosis, and endothelial injury. Chronic inflammation plays a pivotal role in these pathological processes. Elevated SII reflects abnormal immune activation, particularly an increase in neutrophils and platelets. They contribute to endothelial damage, atherosclerosis, and thrombosis (46). Therefore, SII, as an inflammatory marker, holds significant relevance in predicting cardiovascular mortality. Studies indicate that elevated SII is strongly correlated with cardiovascular mortality among individuals with DM. This may be explained by its ability to comprehensively reflect sustained immune system activation. This indicates a higher risk of cardio-vascular events. However, no significant link was observed between SII and CVD or PAD. This apparent inconsistency highlights the limitations of clinical endpoint definitions. Imaging-diagnosed PAD typically represents chronic progressive lesions, while mortality endpoints often reflect acute events (e.g., sudden cardiac death). These acute events are more influenced by inflammation-induced electrophysiological disturbances [50]. These findings imply that SII is more suitable for identifying short-term high-risk conditions rather than long-term structural changes (47).

Although SII demonstrates a robust predictive effect for microvascular complications and mortality, it is not associated with certain outcomes, which needs to be further discussed. For instance, Yang et al. (48) reported no significant correlation between SII and kidney mortality, potentially attributable to the kidney’s unique compensatory mechanisms. Renal tubular epithelial cells mitigate oxidative stress through mitochondrial biogenesis mediated by PGC-1α, partially offsetting inflammation-induced damage (49). Moreover, anti-inflammatory agents (e.g., SGLT2 inhibitors) are extensively used in patients with advanced kidney disease, which may confound the true effect of SII on kidney mortality. However, most existing studies do not perform stratified analyses to explore this issue. In contrast, for non-classical outcomes such as diabetic macular edema (DME), Özata et al. (14) demonstrated an association between SII and disease severity, particularly in advanced-stage patients. This finding may reflect the dual detrimental effects of platelet-mediated microthrombosis and neutrophil elastase-induced disruption of the blood-retinal barrier. However, given a limited number of such studies and their sample sizes, it is imperative to corroborate these findings in larger cohort studies.

### 4.1 Limitation

Despite consistent findings, several limitations warrant cautious interpretation.

First, most included studies were retrospective, which inherently limit the ability to fully control for confounding factors.

Second, SII is calculated from complete blood count parameters, yet blood sampling conditions were not standardized across studies. Crucially, the potential confounding influence of unreported comorbidities (e.g., chronic kidney disease, heart failure, autoimmune diseases) could not be adequately accounted for, potentially affecting both SII and diabetic outcomes and biasing the effect estimates. Future studies should systematically collect comorbidity data to enable adjustment in multivariable models.

Third, the predominance of studies conducted in Asian populations may limit the external validity of the findings. Future research should establish ethnicity-specific predictive models through multicenter collaboration and prospectively validate the clinical utility of SII.

Fourth, this study primarily reflected T2DM populations, thereby limiting generalizability to T1DM. This constraint stems from the scarcity of high-quality studies examining SII’s prognostic value in T1DM populations within existing literature. Future research should further explore SII’s predictive utility specific diabetes subtypes, such as T1DM.

Fifth, the predictive value of SII for any DM-related complication was not assessed, as most studies examined individual complications or specific outcomes. Prospective studies are therefore needed to evaluate major complications collectively, allowing direct assessment of SII’s ability to predict the overall risk of “any complication” in DM patients.

Sixth, substantial variability in SII values across studies prevented standardization of thresholds for SMDs and limited the generalizability of its direct clinical application. Different studies used heterogeneous cutoff values (e.g., median, quartile, optimal Youden index) to define “high” SII, complicating cross-study comparisons and hindering the identification of a universally applicable cutoff. Such discrepancies likely arise from differences in study populations, outcome definitions, and statistical approaches. Future work should establish and validate optimal SII thresholds tailored to specific populations (e.g., by ethnicity, age group) and specific outcomes (e.g., progression of DN, cardiovascular mortality).

Seventh, while this meta-analysis confirms SII’s independent prognostic value, integrating SII into composite scores alongside established clinical parameters (e.g., HbA1c, estimated glomerular filtration rate [eGFR], traditional cardiovascular risk factors) or other inflammatory biomarkers may yield superior predictive performance for diabetic complications and mortality. Future research should develop and validate such integrated risk models to enhance clinical utility.

Eighth, the potential impact of specific glucose-lowering medications (particularly those with anti-inflammatory properties, such as SGLT2 inhibitors or GLP-1 receptor agonists), statins, or detailed glycemic control patterns (e.g., HbA1c variability) on SII-outcome associations could not be assessed due to insufficient reporting in the included studies. This remains an important area for future investigation.

Finally, the extreme heterogeneity observed in some analyses (I²>90%) substantially compromises the confidence of pooled estimates. Although subgroup analyses were implemented, the sources of heterogeneity could not be fully elucidated due to limited data and the absence of meta-regression. Establishing standardized methodological protocols is imperative in subsequent research to reduce heterogeneity.

Together, these limitations suggest that the current conclusions regarding SII’s prognostic value require further validation in future high-quality research.

## 5 Conclusion

This study highlights that SII, as a comprehensive inflammatory marker, effectively reflects systemic immune responses and is closely associated with diabetic nephropathy, retinopathy, all-cause mortality, and cardiovascular mortality. Future research should precisely define the application scenarios of SII, particularly in DM patients across different age groups and disease stages. Additionally, combining SII with other inflammatory markers and biological information to develop dynamic predictive models may enhance the accuracy of predicting diabetic complications and mortality, thereby paving the way for personalized medicine and precision treatment.

## Data Availability

The original contributions presented in the study are included in the article/**Supplementary Material**. Further inquiries can be directed to the corresponding author.

## Supplementary material

Supplementary Figure 1 Subgroup analysis of SII and DN. **(A)** Subgroup analysis by age; **(B)** Subgroup analysis by sample size; **(C)** Subgroup analysis by region.

Supplementary Figure 2 Subgroup analysis of SII and DR. **(A)** Subgroup analysis by age; **(B)** Subgroup analysis by SII cut-off; **(C)** Subgroup analysis by sample size; **(D)** Subgroup analysis by region.
