# Supplementary figures and images for: Predictive value of systemic immune-inflammation index in patients with diabetes mellitus: a systematic review and meta-analysis
Source: Front Endocrinol (Lausanne). 2025 Sep 23;16:1617814. doi: 10.3389/fendo.2025.1617814 (PMC12500460; doi:10.3389/fendo.2025.1617814)

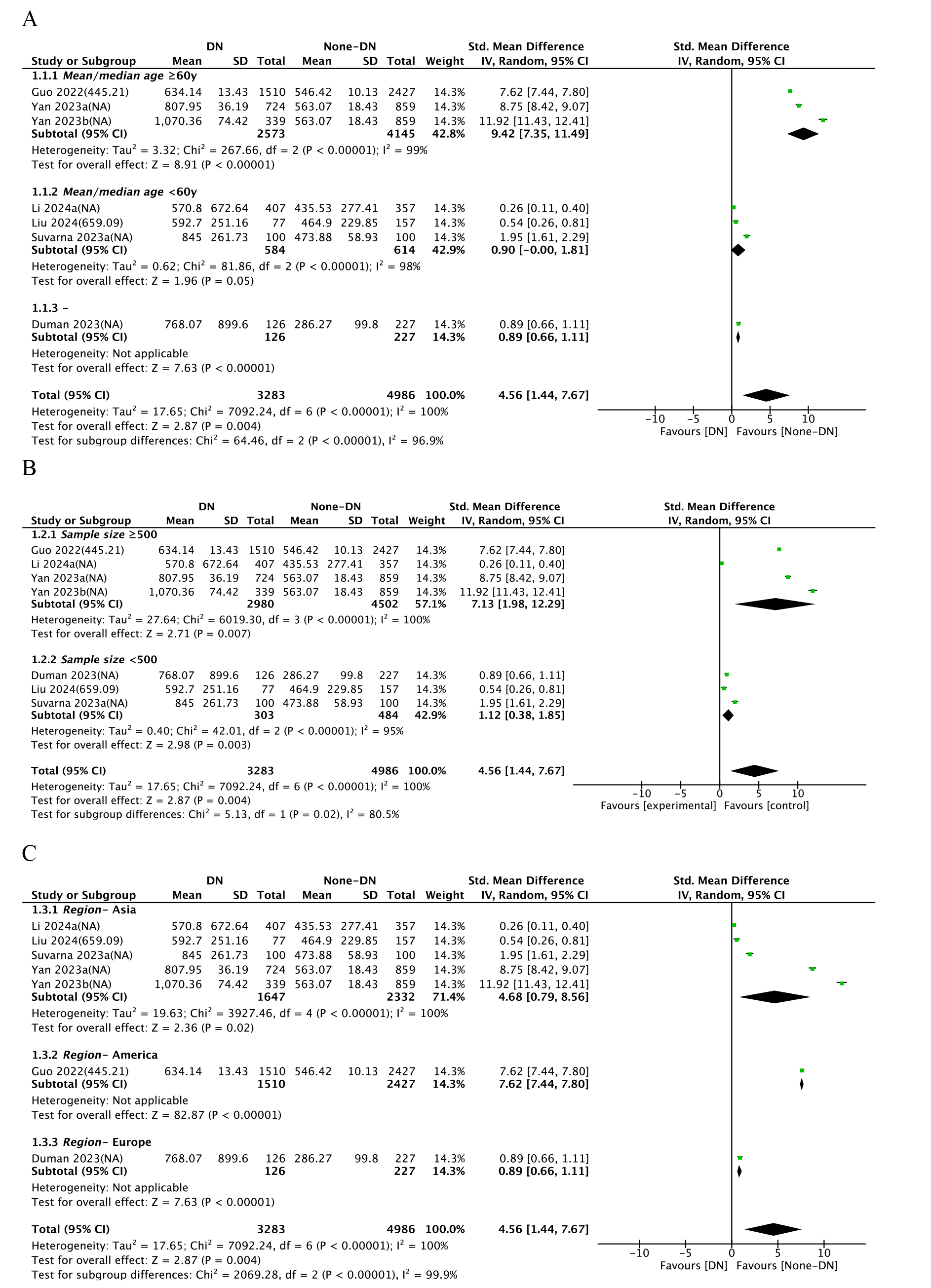

Supplement: Supplementary Figure 1 — Subgroup analysis of SII and DN. (A) Subgroup analysis by age; (B) Subgroup analysis by sample size; (C) Subgroup analysis by region. [file Image1.tif]

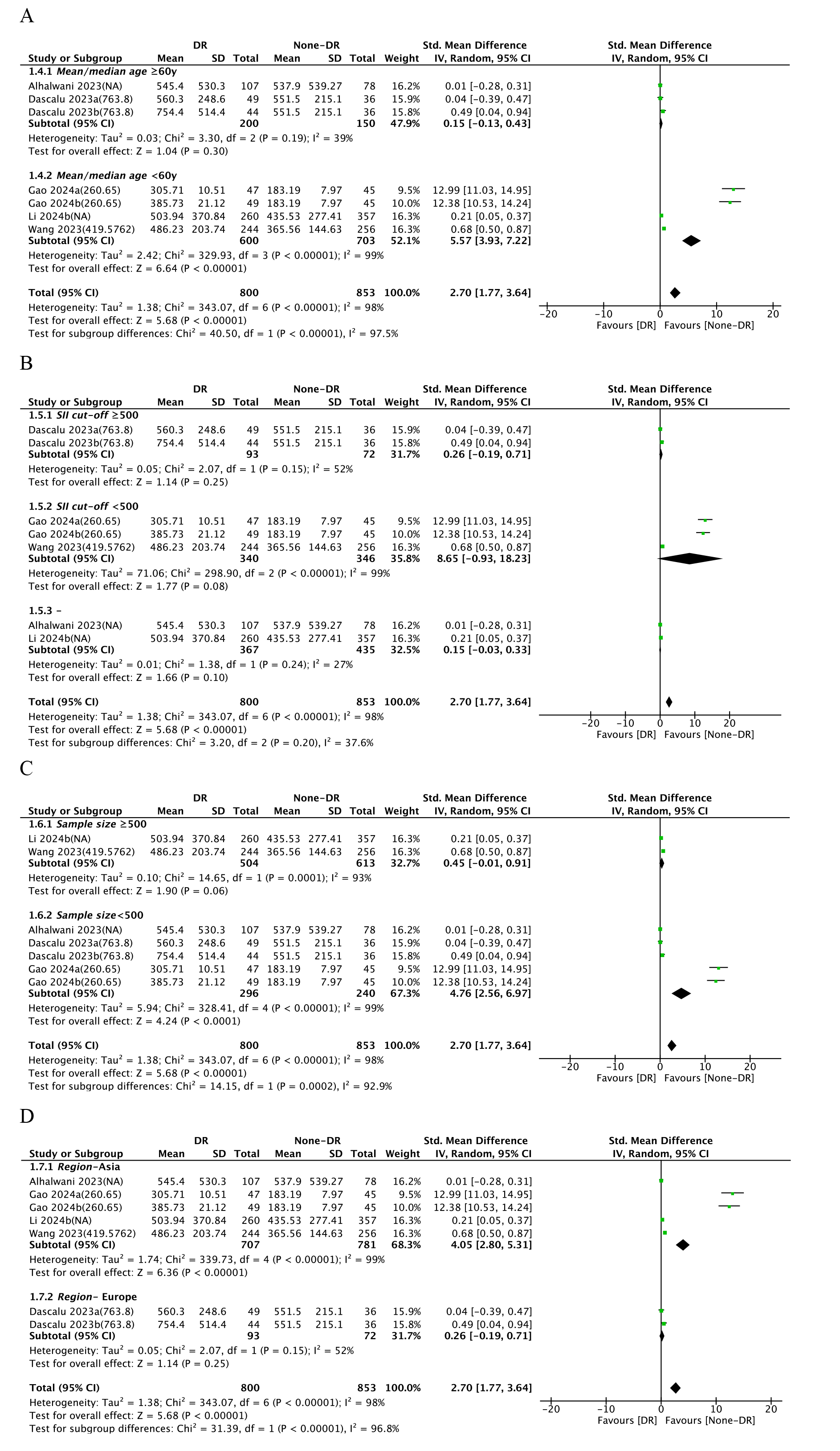

Supplement: Supplementary Figure 2 — Subgroup analysis of SII and DR. (A) Subgroup analysis by age; (B) Subgroup analysis by SII cut-off; (C) Subgroup analysis by sample size; (D) Subgroup analysis by region. [file Image2.tif]
