# Supplementary material for: Predictive value of systemic immune-inflammation index in patients with diabetes mellitus: a systematic review and meta-analysis
Source: Front Endocrinol (Lausanne). 2025 Sep 23;16:1617814. doi: 10.3389/fendo.2025.1617814 (PMC12500460; doi:10.3389/fendo.2025.1617814)
Supplement: Supplementary file 3 [file Table1.docx]

Table S1 The search strategy

Pubmed-336

(("Diabetes Mellitus"[Mesh]) OR ((Diabetes) OR (DM))) AND ((systemic immune inflammation index) OR (SII))

Embase-466


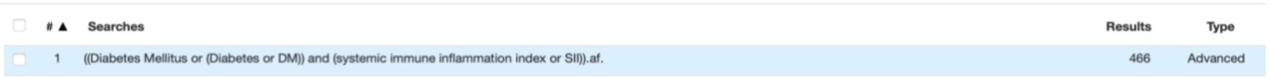


Cochrane-15


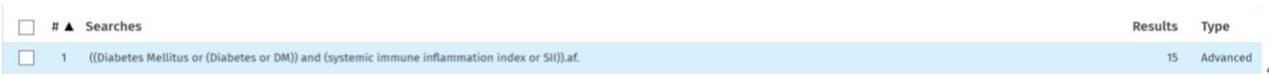


WOS-292

((Diabetes Mellitus) OR ((Diabetes) OR (DM))) AND ((systemic immune inflammation index) OR (SII)) (Topic)
