# Supplementary material for: Predictive value of systemic immune-inflammation index in patients with diabetes mellitus: a systematic review and meta-analysis
Source: Front Endocrinol (Lausanne). 2025 Sep 23;16:1617814. doi: 10.3389/fendo.2025.1617814 (PMC12500460; doi:10.3389/fendo.2025.1617814)
Supplement: Supplementary file 4 [file Table2.docx]

Table S2 Detailed characteristics of included studies

| **Author+year** | **study period** | **region** | **study design** | **Population^1^** | **No. of patients** | **Gender** | | **Age** | **BMI** | **HbA1c** | **SII cut-off^2^** | **outcome** | **Quality**  **score** |
| --- | --- | --- | --- | --- | --- | --- | --- | --- | --- | --- | --- | --- | --- |
|  |  |  |  |  |  | **Male** | **Female** |  |  |  |  |  |  |
| Alhalwani(21) 2023 | 2018-2020 | Saudi Arabia | Retrospective | T2DM | 185 | 80 | 105 | 60.02 | NA | 8.91 | NA | DR | 7 |
| Chen(24) 2023a | 1999–2018 | United States | Prospective | T2DM | 8668 | 4459 | 4209 | 59.19 | NA | 8.43 | NA | Cardiovascular mortality | 7 |
| Chen(24) 2023b | 1999–2018 | United States | Prospective | T2DM | 8668 | 4459 | 4209 | 59.19 | NA | 8.43 | NA | All-cause mortality | 7 |
| Dascalu(10) 2023a | 2022.1-2022.12 | Romania | Retrospective | T2DM | 129 | 67 | 62 | 65.6 | NA | 7.6 | 763.8 | Non-proliferative DR | 7 |
| Dascalu(10) 2023b | 2022.1-2022.12 | Romania | Retrospective | T2DM | 129 | 67 | 62 | 65.6 | NA | 7.6 | 763.8 | Proliferative DR | 7 |
| Duman(17) 2023 | 2020.3-2022.12 | Turkey | Retrospective | T2MD | 539 | 297 | 242 | NA | NA | NA | NA | DN | 7 |
| Fajkic(31) 2024 | NA | bosnia and herzegovina | Prospective | T2DM | 80 | 40 | 40 | 48.58 | 24.55 | NA | 408.57 | Metabolic syndrome | 7 |
| Gao(8) 2024a | 2015.1-2022.1 | China | Retrospective | T2DM | 141 | 78 | 63 | 52.82 | NA | 7.44 | 260.65 | Non-proliferative DR | 7 |
| Gao(8) 2024b | 2015.1-2022.1 | China | Retrospective | T2DM | 141 | 78 | 63 | 52.82 | NA | 7.44 | 260.65 | Proliferative DR | 7 |
| Guo(9) 2022 | 2011-2018 | United States | Retrospective | T2MD | 3937 | 2040 | 1897 | 60.51 | 33.1 | NA | 445.21 | DN | 7 |
| Li(22) 2024a | 2020–2023 | China | Retrospective | DM | 1058 | 337 | 721 | 54.67 | 26.04 | 8.59 | NA | DN | 6 |
| Li (22) 2024b | 2020–2023 | China | Retrospective | DM | 1058 | 337 | 721 | 54.67 | 26.04 | 8.59 | NA | DR | 6 |
| Li(22) 2024c | 2020–2023 | China | Retrospective | DM | 1058 | 337 | 721 | 54.67 | 26.04 | 8.59 | NA | PAD | 6 |
| Li(25) 2024 | 1999-2020 | United States | Prospective | DM | 983 | 496 | 486 | 69.68 | NA | NA | NA | All-cause mortality | 7 |
| Liu(23) 2024 | 2016.1-2018.12 | China | Retrospective | T2DM | 234 | 157 | 77 | 59 | 24.06 | 8.76 | 659.09 | DN | 7 |
| Mariaca(32) 2024 | NA | Spain | Retrospective | T1DM | 602 | 318 | 284 | 48.7 | 26.3 | 7.6 | NA | CVD | 7 |
| Meng(26) 2024a | 2005-2016 | United States | Retrospective | DM | 4972 | 2576 | 2396 | 59.03 | 31.49 | 7.05 | 983.5714 | Cardiovascular mortality | 7 |
| Meng(26) 2024b | 2005-2016 | United States | Retrospective | DM | 4972 | 2576 | 2396 | 59.03 | 31.49 | 7.05 | 983.5714 | All-cause mortality | 7 |
| Muresan(30) 2023 | 2020.1-2023.3 | Romania | Retrospective | T2DM | 198 | 93 | 105 | 64.36 | 29.32 | 6.83 | 615.91 | CVD | 7 |
| Özata Gündoğdu (14)2022 | 2015-2020 | Turkey | Retrospective | DM | 120 | 58 | 62 | 56.5 | NA | NA | NA | serous macular detachment secondary to diabetic macular edema | 9 |
| Song(15) 2023 | 2020.6-2022.9 | China | Retrospective | T2DM | 434 | 245 | 188 | NA | NA | NA | NA | PAD | 7 |
| Suvarna(16) 2023a | 2021.1-2021.6 | India | Retrospective | T2DM | 300 | 125 | 175 | 57.96 | NA | NA | NA | DN | 6 |
| Suvarna(16) 2023b | 2021.1-2021.6 | India | Retrospective | T2DM | 300 | 125 | 175 | 57.96 | NA | NA | NA | CVD | 6 |
| Tang(27) 2024a | 1999-2018 | United States | Prospective | DM | 45454 | 22532 | 22922 | 47.35 | 28.73 | 5.6 | 963.1 | Cardiovascular mortality | 7 |
| Tang(27) 2024b | 1999-2018 | United States | Prospective | DM | 45454 | 22532 | 22922 | 47.35 | 28.73 | 5.6 | 963.1 | All-cause mortality | 7 |
| Wang(18) 2023 | 2021.1-2023.6 | China | Retrospective | T2DM | 500 | 343 | 157 | 59 | 26 | 8.55 | 419.5762 | DR | 7 |
| Yan(19) 2023a | 2012.8-2015.9 | China | Retrospective | T2DM | 1922 | 975 | 947 | 60.72 | 24.36 | 9.3 | NA | DN stages 1–2 Alb | 7 |
| Yan(19) 2023b | 2012.8-2015.9 | China | Retrospective | T2DM | 1922 | 975 | 947 | 60.72 | 24.36 | 9.3 | NA | DN stage 3 Alb+ DN -non-Alb | 7 |
| Yan(19) 2023c | 2012.8-2015.9 | China | Retrospective | T2DM | 1922 | 975 | 947 | 60.72 | 24.36 | 9.3 | NA | DN | 7 |
| Yang(28) 2023a | 1999-2018 | United States | Prospective | T2DM | 8697 | 4475 | 4222 | 58.95 | NA | NA | 702.6 | Kidney mortality | 7 |
| Yang(28) 2023b | 1999-2018 | United States | Prospective | T2DM | 8697 | 4475 | 4222 | 58.95 | NA | NA | 702.6 | Cardiovascular mortality | 7 |
| Yang(28) 2023c | 1999-2018 | United States | Prospective | T2DM | 8697 | 4475 | 4222 | 58.95 | NA | NA | 702.6 | All-cause mortality | 7 |
| Yilmaz(20) 2023 | 2016-2022 | Turkey | Retrospective | DM | 231 | 140 | 91 | 72.52 | NA | NA | NA | Mortality after below-knee amputation | 7 |
| Zhang(29) 2024a | 2005-2018 | United States | Prospective | DM | 6412 | 3332 | 3080 | 59.36 | NA | NA | 692.13 | Cardiovascular mortality | 7 |
| Zhang(29) 2024b | 2005-2018 | United States | Prospective | DM | 6412 | 3332 | 3080 | 59.36 | NA | NA | 692.13 | All-cause mortality | 7 |

1. The study using "DM" included a mixed population and did not clearly distinguish between T1DM and T2DM.

2. The original study determined the optimal threshold of SII for predicting prognosis in patients with diabetes based on the receiver operating characteristic (ROC) curve.

DR, Diabetic retinopathy.

DN, Diabetic nephropathy.

CVD, Cardiovascular disease.

PAD, Peripheral artery disease.

DM, Diabetes Mellitus.

T1DM, type 1 Diabetes Mellitus.

T2DM, type 2 Diabetes Mellitus.

SII, systemic immune-inflammatory index.

NA, Data not reported in the source manuscript.
